# Circumcision-related tragedies seen in children at the Komfo Anokye Teaching Hospital, Kumasi, Ghana

**DOI:** 10.1186/s12894-016-0183-1

**Published:** 2016-11-08

**Authors:** Kwaku Addai Arhin Appiah, Christian Kofi Gyasi-Sarpong, Roland Azorliade, Ken Aboah, Dennis Odai Laryea, Kwaku Otu-Boateng, Kofi Baah-Nyamekye, Patrick Opoku Manu Maison, Douglas Arthur, Isaac Opoku Antwi, Benjamin Frimpong-Twumasi, Edwin Mwintiereh Yenli, Samuel Kodzo Togbe, George Amoah

**Affiliations:** 1 Department of Surgery, Komfo Anokye Teaching Hospital, Kumasi, Ghana; 2 Department of Surgery, School of Medical Sciences-KNUST, Kumasi, Ghana; 3 Public Health Unit, Komfo Anokye Teaching Hospital, Kumasi, Ghana; 4 Department of Surgery, Tamale Teaching Hospital, Tamale, Ghana

**Keywords:** Circumcision, Penile amputation, Circumcision injury, Urethrocutaneous fistula, Ghana

## Abstract

**Background:**

Circumcision is a common minor surgical procedure and it is performed to a varying extent across countries and religions. Despite being a minor surgical procedure, major complications may result from it. In Ghana, although commonly practiced, circumcision-related injuries have not been well documented. This study is to describe the scope of circumcision-related injuries seen at the Komfo Anokye Teaching Hospital in Kumasi, Ghana.

**Methods:**

The study was conducted at the Urology Unit of the Komfo Anokye Teaching Hospital in Kumasi. Consecutive cases of circumcision-related injuries seen at the unit over an 18 month period were identified and included in the study. Data was collected using a structured questionnaire. Data was entered and analysed using SPSS version 16. Charts and tables were generated using Microsoft Excel.

**Results:**

A total of 72 cases of circumcision-related injuries were recorded during the 18 month period. Urethrocutaneous fistula was the commonest injury recorded, accounting for 77.8 % of cases. Other injuries recorded were glans amputations (6.9 %); iatrogenic hypospadias (5.6 %), and epidermal inclusion cysts (2.8 %). The majority of children were circumcised in health facilities (75 %) and nurses were the leading providers (77.8 %). The majority of circumcisions were conducted in the neonatal period (94.7 %).

**Conclusion:**

Circumcision-related injuries commonly occurred in the neonatal period. Most of the injuries happened in health facilities. The most common injury recorded was urethrocutaneous fistula but the most tragic was penile amputation. There is the need for education and training of providers to minimise circumcision-related injuries in Ghana.

## Background

Circumcision is routinely performed in most parts of Ghana as a tradition. While generally regarded as a minor surgical procedure, major complications may result from it [1–4]. Although circumcision injuries are unintended, the prominence of circumcision as a cause of major injury in children is not recognised, as the world report on injury in children did not identify circumcision-related injuries as significant causes of injury-related morbidity and mortality in children [5]. This notwithstanding, some circumcision injuries may be associated with long term social and psychological challenges including the inability to have a fulfilling sexual life as the case may be in penile amputations [6] and even death in some cases of severe haemorrhage [6, 7]. In Nigeria, circumcision-related injuries have been on the ascendancy with an estimated 20 % circumcisions resulting in one form of complication or the other [3]. Various degrees of circumcision-related injuries occur. However, severe ones seldom occur in developed countries [2] where circumcision is practised by well-trained personnel [8]. Circumcision injuries have been associated with all the methods of circumcisions [1, 4] especially in untrained hands [3, 6–8]. In Ghana, data on circumcision-related injuries is scanty. This cross-sectional observational study was designed to describe the scope of circumcision-related injuries seen at the Komfo Anokye Teaching Hospital in Kumasi, Ghana.

## Methods

The study was conducted at the Urology Unit of the Directorate of Surgery, Komfo Anokye Teaching Hospital (KATH). KATH is a major referral centre for the middle and northern zones of Ghana.

All male children below 18 years of age referred to the Komfo Anokye Teaching Hospital’s Urology Unit for treatment of early and late complications of circumcision as determined by our eligibility criteria were included in the study. Urologists at the unit conducted penile examinations and assigned eligible patients specific injury categories as haemorrhage, urethrocutaneous fistula, penile amputation, iatrogenic hypospadias, skin bridges, excess foreskins, epidermal inclusion cysts, buried penis or any other injury that was deemed to be as a result of circumcision. Guardians/parents of eligible children were approached for inclusion in the study. The aim of the study was explained to them and informed consent obtained. Ethical approval was obtained from the Committee on Human Research, Publications and Ethics of the Kwame Nkrumah University of Science and Technology and the Komfo Anokye Teaching Hospital. Data collection involved a structured questionnaire administered by a trained research assistant. Data collected included demographic information, place of circumcision, person circumcising, age at circumcision and clinical examination findings. Data was collected over an 18 month period from September 2012 to February 2014.

Data was entered into SPSS version 16 and the same was utilized for statistical analysis. Microsoft Excel was used to generate the tables and charts.

## Results

A total of 72 cases of children with circumcision-related complications were seen during the 18 month period. The youngest case was recorded in a 2-day old neonate and the oldest case recorded was in an 11-year-old boy. The majority of the children were resident in urban communities (54.0 %).

Over 87 % of children in this study were circumcised before they were 2 weeks old. Only 5.6 % were circumcised after 4 weeks of age (Table 1).

**Table 1:** Age at circumcision for circumcision-related injuries recorded in Kumasi

Age at Circumcision	Frequency	%
≤1 week	34	47.2
>1–2 weeks	29	40.2
>2–3 weeks	3	4.2
>3–4 weeks	2	2.8
>4 weeks	4	5.6
Total	72	100.0

**Fig. 1:**
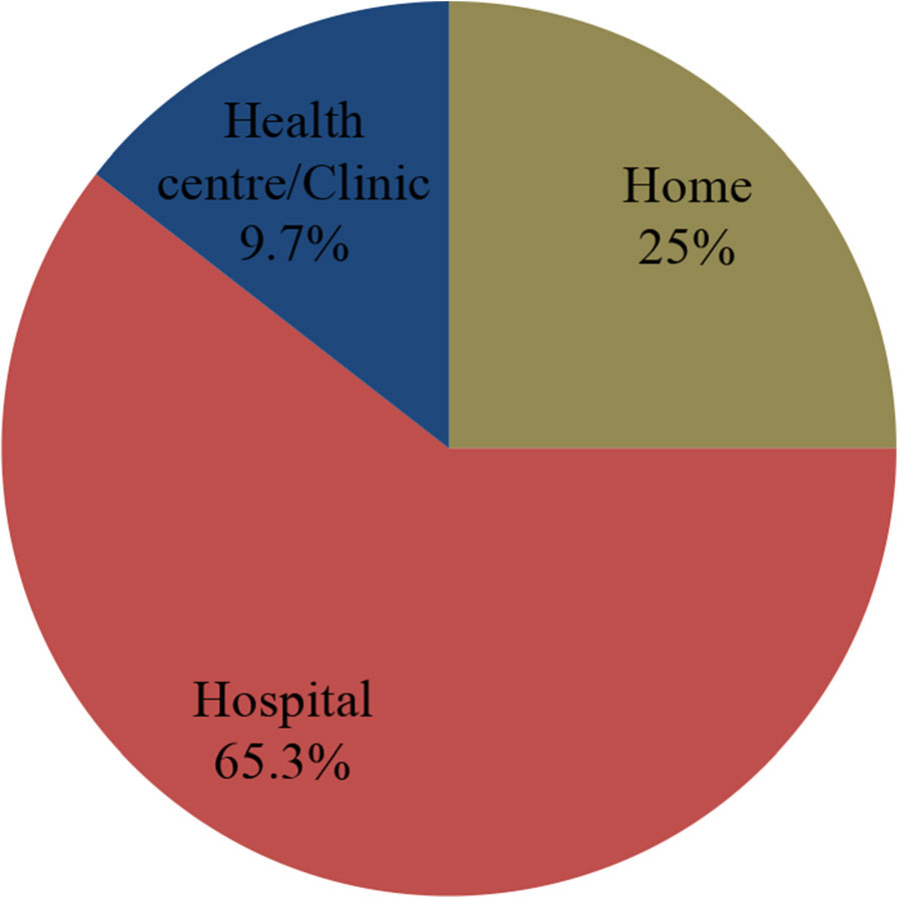
Place of circumcision among children with circumcision-injuries in Kumasi

The majority of children were circumcised in a hospital (65.3 %). The place of circumcision is as shown in Fig. 1. Nurses accounted for the majority of circumcision-related injuries recorded in this study, 77.8 %. Doctors and traditional circumcisers (Wanzams) accounted for 8.3 and 20.8 % of circumcision-related injuries respectively. None of the children seen during the period under review reported within 24 h of injury. The majority of injuries (80.5 %) were seen within 2 weeks of circumcision. Twelve (16.7 %) cases presented within 3 months of circumcision and the remaining 2.8 % presented more than a year after circumcision.

**Fig. 2:**
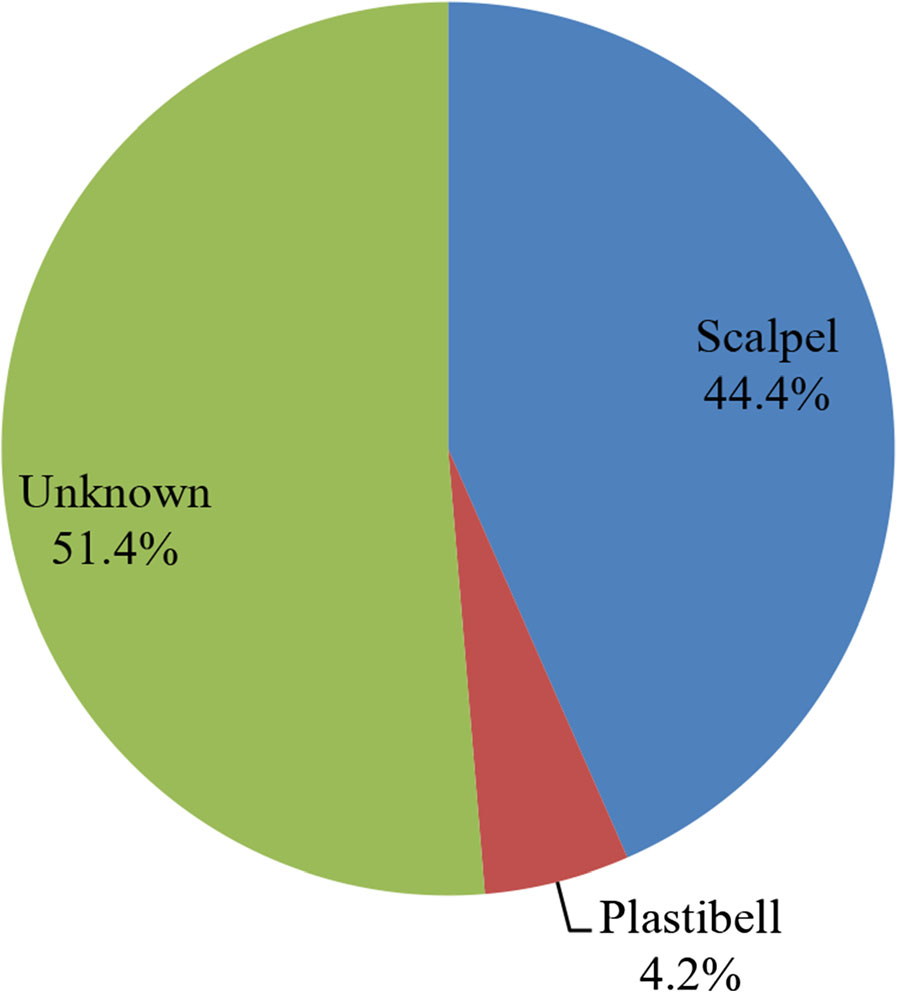
Method of circumcision among cases of circumcision-related injuries recorded in Kumasi

In 37 (51.4 %) of the cases studied, the exact method of circumcision could not be ascertained from the parents of affected children. Figure 2 details the methods of circumcision as recorded among cases seen during this study.

### Complications

The commonest complication recorded in this study was urethrocutaneous fistula (77.8 %). The various categories of complications recorded in this study are as shown in Table 2. There were five cases of glans penis amputations accounting for 6.9 % of complications recorded. Three (60 %) were complete amputations of the glans penis, with the remaining 40 % being partial amputations.

**Table 2:** Categories of complications among children with circumcision-related injuries recorded in Kumasi

Type of Complication	Frequency	Percentage
Urethrocutaneous fistula	56	77.8
Complete Penile Amputation	3	4.1
Iatrogenic Hypospadias	4	5.6
Epidermal Inclusion cyst	3	4.1
Partial Penile Amputation	2	2.8
Skin Bridges	2	2.8
Excess Foreskin	2	2.8
Total	72	100.0

## Discussion

Circumcision remains one of the oldest and commonest surgical procedures performed on young boys worldwide [9, 10]. It is widely practiced in the United States [2] and especially in Israel where virtually every male child is circumcised [11]. However in Europe it is rarely performed [12]. The notable advantages of circumcision include: reduction in early childhood urinary tract infections, which is also noted in adult men [13–17], reduction of HIV transmission by almost 60 % [18–21], and reduction in the incidence of penile cancer [22–24]. Ghana’s circumcision rate is estimated to be on the high side as the majority of ethnic groups and religions identify circumcision as an appropriate religious or cultural practice for males to undergo [7].

The timing of circumcision among children in this study suggests an early age of circumcision in Ghana as over 87 % of cases had circumcision done in the neonatal period (Table 1). This is similar to findings by Osifo and Oraifo [7] and Chaim et al. [11]. However, in Eastern and Southern Africa and some parts of the pacific, circumcision is performed far beyond the neonatal period [6, 25, 26]. For some, it is a rite of passage into adulthood [6, 25]. Only 5.6 % of our cases were circumcised beyond the neonatal period. While our study population may not be representative of the population of Ghana, it provides an indication that most circumcisions are being performed during the neonatal period. This may have implications for interventions in the areas of circumcision such as persons to target for training, timing of educational messages on circumcision and the location of circumcision services.

In this study, over 65 % of children with the complications recorded had their circumcisions done in a hospital. The proportion is even higher (75 %) when lower level health facilities (health centres and clinics) are included. Similarly in Nigeria, more circumcisions were done in orthodox medical centres (66.9 %) than traditional settings (33.1 %) [27]. Our findings however, contrast sharply with studies from Southern and Eastern Africa where virtually all circumcisions are performed outside hospitals as part of traditional rites of passage into manhood with high complication rates [6, 25]. In Israel, however, although a significant proportion of circumcisions are undertaken outside health facilities by the ritual circumciser, a lower proportion of complications have been recorded because they are well trained and the practice is regulated [11].

In this study, nurses accounted for the majority of circumcision-related injuries - 77.8 % of cases. Doctors and traditional circumcisers (locally referred to as Wanzams) accounted for 8.3 and 20.8 % of the circumcision-related complications respectively. Likewise in Nigeria, nurses were found to account for the majority of complications (55.9 %) with doctors and traditional circumcisers accounting for 35.1 and 9 % respectively [3]. In a comparative study by Atikeler et al., it was found that circumcisions done by unlicensed circumcisers resulted in more early phase complications as well as late lifelong complications compared with licensed surgeons [8]. Even among physicians performing circumcisions, there is evidence that there a is lack of formal training amongst them as to how to perform circumcision correctly and providers also lack the requisite skills to manage the complications of circumcisions [4, 7]. Our findings indicate gaps either in knowledge and/or practice among persons providing circumcision services in health facilities in Ghana and it is therefore imperative that training workshops are organised for all providers especially nurses to reduce the incidence of circumcision-related injuries in the future.

The method of circumcision was unknown in 37 (51.4 %) of the cases. A significant proportion (91.4 %) of the cases for which the method of circumcision was known underwent surgical circumcision with a scalpel and this is still consistent with other studies that have examined circumcision-related injuries and complications in the West African sub-region [3, 7]. Due to the high proportion (51.4 %) of cases for which the method of circumcision was unknown in this study, we are unable to associate the method of circumcision with the complications observed. However, there is evidence that the Plastibel device poses a higher risk of complication compared with conventional dissection [1].

**Fig. 3:**
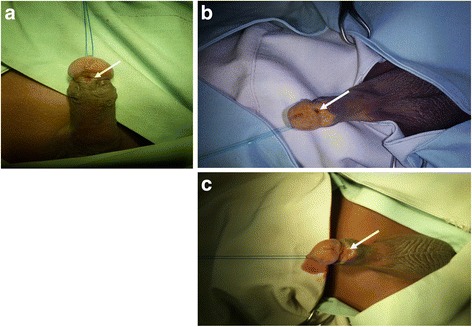
**a** A small (<5 mm) sized urethrocutaneous fistula (*arrowed*). **b** Medium sized (5–10 mm) urethrocutaneous fistula (*arrowed*). **c** Large sized (>10 mm) urethrocutaneous fistula (*arrowed*)

The majority of our cases (77.8 %) had urethrocutaneous fistulae. Urethrocutaneous fistulae have largely been associated with hypospadias repair in developed countries [28, 29] and not circumcision. The proportion of urethrocutaneous fistulae recorded in this study contrasts sharply with findings in Nigeria by Osifo and Oraifo et al. in which urethrocutaneous fistula accounted for only 21 % of complications recorded [7] and that of Okeke et al., where no fistula was recorded [3]. The urethrocutaneous fistulae in the present study ranged in sizes from pinhole defects (<5 mm) to very big defects (>10 mm) on the ventral aspect of the glans penis (Fig. 3a-c). We think the management of haemorrhage/bleeding during circumcision may be accounting for the high numbers of urethrocutaneous fistulae observed. The ligation of bleeding sites with larger-sized sutures and direct laceration into the urethra during circumcision may be responsible for the high numbers of urethrocutaneous fistulae observed in this study [30]. The occurrence of urethrocutaneous fistula has also been associated with the Plastibel device [28].

**Fig. 4:**
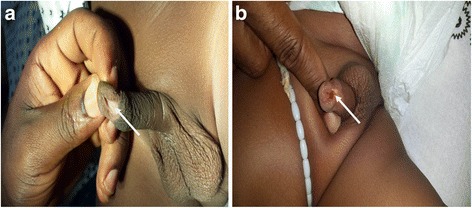
**a** Iatrogenic hypospadias (*arrowed*). **b** Iatrogenic hypospadias (*arrowed*)

There were four cases (5.6 %) of iatrogenic hypospadias (Fig. 4a-b). This is one of the worse forms of circumcision-related injuries. Complete ligation of the artery to the frenulum may cause extensive tissue necrosis on the ventrum of the glans penis leading to the iatrogenic hypospadias [30]. Isolated cases of iatrogenic hypospadias have been reported after the circumciser performed a ventral rather than a dorsal slit prior to the start of circumcision. It is imperative that the proper plane is entered into for the initial separation of adhesions so that the meatus is not inadvertently entered into, and then damaged [2].

The iatrogenic hypospadias seen in this study may not necessarily be as a result of complications of circumcision but may have been missed mega meatus with intact prepuce variants before circumcision and only found thereafter. This study is unable to determine whether the iatrogenic hypospadias observed had megameatus with intact prepuce before circumcision. Clinically, these are difficult to distinguish after circumcision [2].

**Fig. 5:**
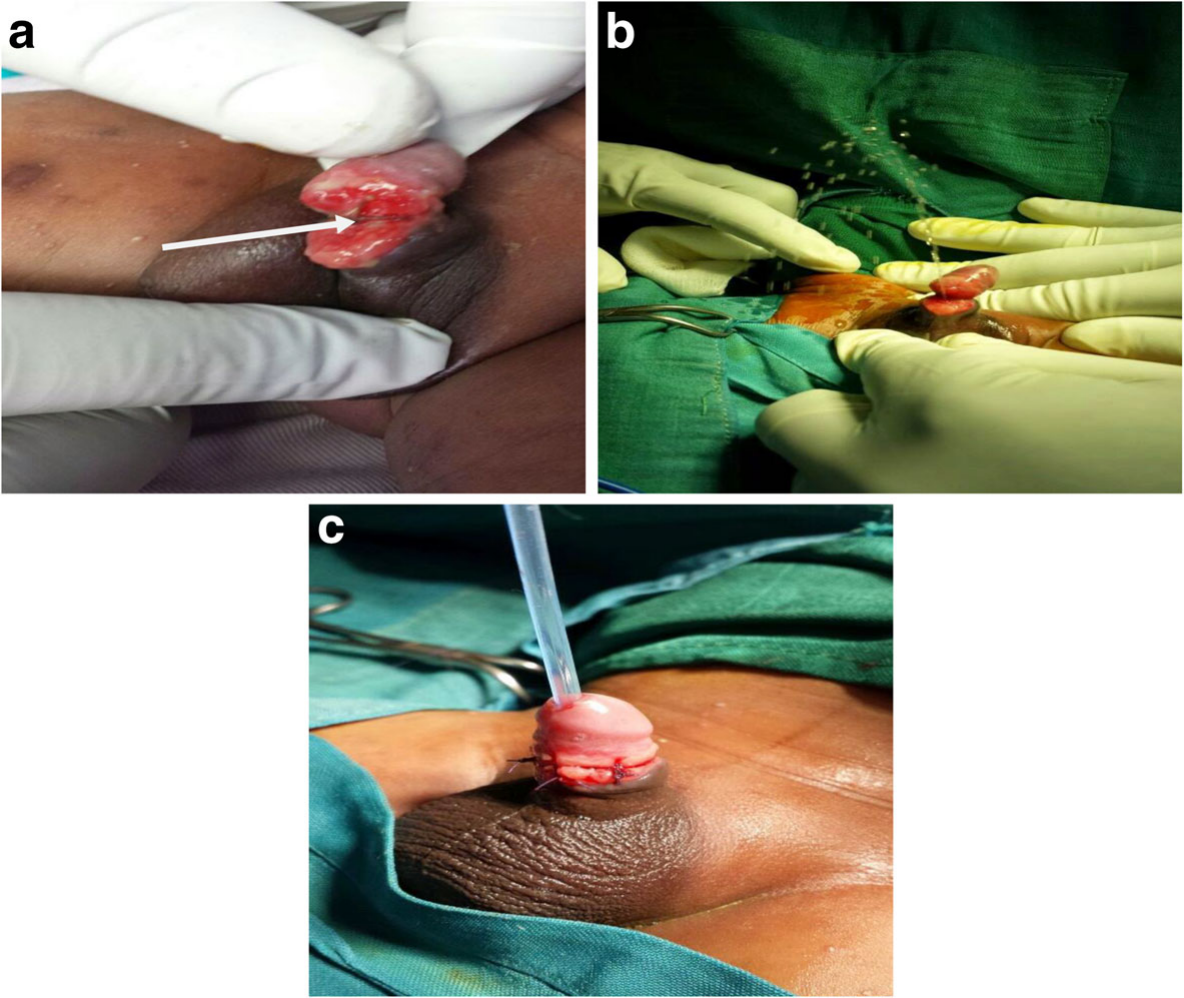
**a** Partial penile amputation from tourniquet effect of a suture material (*arrowed*) seen within 48 h. **b** Patient urinating immediately after release of tourniquet. **c** Immediate post-repair

**Fig. 6:**
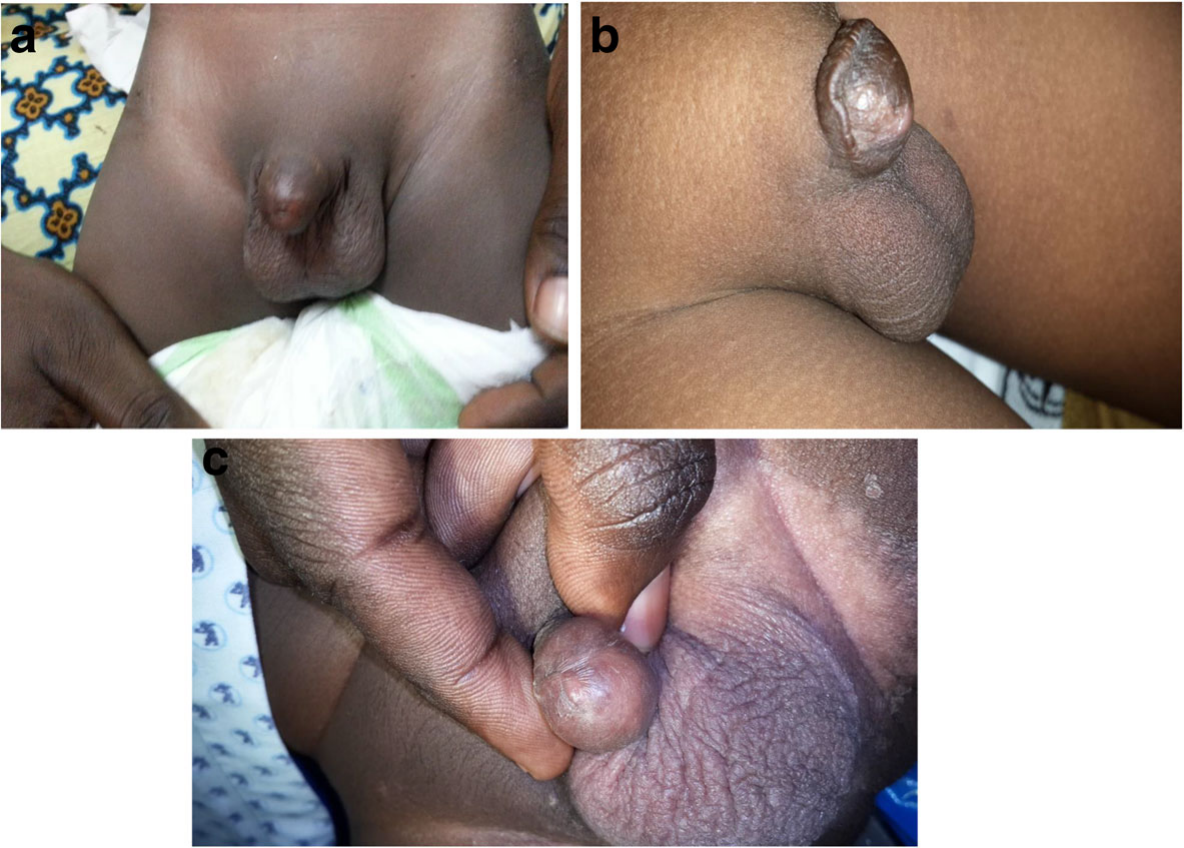
**a** Complete glans penis amputation seen 3 years post circumcision with scarred stump end. **b** near total penile amputation seen 2 years post circumcision. **c** Complete glans penis amputation from plastibel circumcision seen 3 months post circumcision with meatal occlusion

**Fig. 7:**
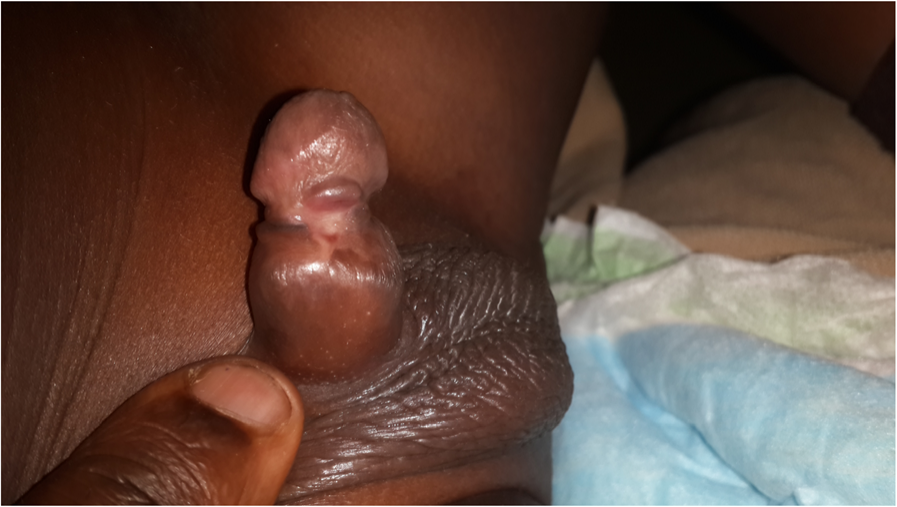
Healed partial penile amputation from tourniquet effect with a constriction ring and urethrocutaneous fistula

The most tragic form of circumcision-related injury is penile amputation and it was the second leading complication recorded in this study, accounting for 6.9 % overall. Complete penile amputation accounted for 4.1 % of all complications. This is higher compared with the 3.1 % recorded in Nigeria by Okeke et al. [3] but lower than the 8 % recorded in a study in Turkey by Ceylan et al. [31]. One case of partial penile amputation recorded in this study was reported within 48 h and this was repaired successfully (Fig. 5a-c). However, the other case reported after six months and presented with a healed wound with a constriction band and urethrocutaneous fistula (Fig. 6). In all the cases of complete penile amputations, the parents of the babies were falsely reassured that all was well by the circumcisers either because of ignorance on their part or for fears of litigation against them. As a result they all presented late with difficulty passing urine as wound healing with scarring at the stump ends caused meatal stenosis (Fig. 7a-c). Penile glans amputation like many others is a preventable complication of circumcision if proper attention is paid to detail and the circumcision is carried out by properly trained personnel [32–34]. Again if the practitioners were trained to recognise these complications, they would have referred such patients immediately with the severed penile tissue properly preserved on ice so that penile reattachment could be attempted. This may have resulted in better cosmetic outcomes for such patients [32, 35, 36].

**Fig. 8:**
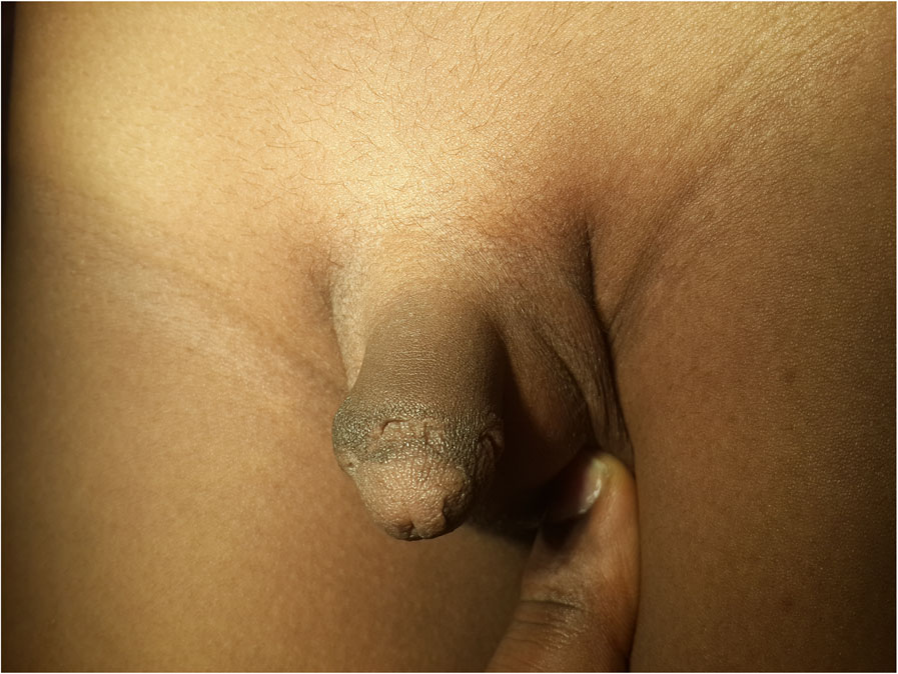
Skin bridges in an 8year old boy

**Fig. 9:**
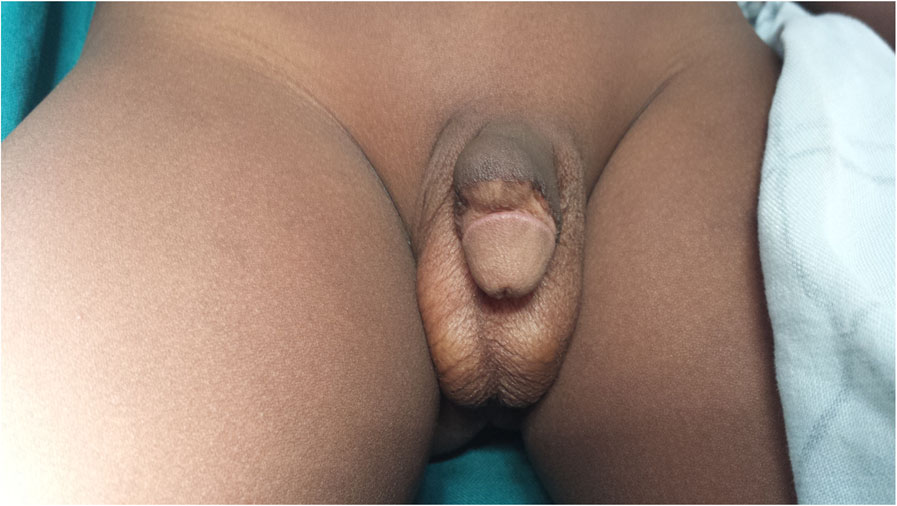
Epidermal inclusion cyst seen 7 months post circumcision

There were four cases of epidermal inclusion cysts (Fig. 8) with the youngest aged 7 months presenting with a painless swelling on the dorsum of the penis. Epidermal inclusions cysts are known to result from the implantation of skin in the subcutaneous tissue during circumcision [37]. They are considered rare in some countries [36, 37]. Our findings may suggest that these may not be rare. They are known to be usually asymptomatic and may not be reported unless issues bordering on aesthetics or pain from infection emerge [37]. Skin bridges (Fig. 9) are also recognised minor complications of circumcision and are easily treated [38]. They may go unnoticed unless cosmetic issues or pain and infection occur. In our study, these two categories of complications each accounted for 2^.^8 % of all complications. There were two cases of excess foreskin. This results from inadequate excision of the foreskin. The parents brought them because they were dissatisfied with the cosmetic appearance of the penis. In other studies, excess foreskin constituted the predominant late complication of circumcision [11].

Our study did not record any case of haemorrhage which was among the leading complications recorded by Gee and Ansell [39]. Haemorrhage, most likely, will occur in the first few hours of circumcision. We surmise that late reporting may account for the non-recording of haemorrhage as a complication in our study. It may also be due to clients accessing acute care in lower level health facilities and only reporting severe complications to the Urology Unit. This may also imply that the complete spectrum of circumcision-related injuries may not have been fully covered in our study, thus a bigger burden may exist.

Circumcision has social, cultural and religious implications and this may account for the high uptake of the procedure despite the associated complications [1, 6, 8]. It is imperative that the procedure is made safe in order to ensure that children undergoing the procedure in the future do not develop complications. Persons who have not been circumcised have been ostracised in some parts of Africa; this can take the form of denial of marriage since uncircumcised men were frowned upon [6, 35, 36] in the past and such stereotypes may still exist.

## Conclusion

Neonatal circumcision, a common practice in Ghana is associated with several and sometimes tragic complications such as penile amputations. The high proportion of urethrocutaneous fistulae recorded in this study requires further investigation to determine the underlying causes and allow for the institution of appropriate preventive measures. There is the need for further studies focusing on the immediate or early complications following circumcision including injuries related to specific methods of circumcision. The training of providers in order to reduce the incidence of injuries is also recommended.

## Abbreviations

HIV: Human Immunodeficiency virus
KATH: Komfo Anokye Teaching Hospital
SPSS: Statistical Software Package for Social Sciences
